# Disorder of Sexual Development and Congenital Heart Defect in 47XYY: Clinical Disorder or Coincidence?

**DOI:** 10.1155/2015/802162

**Published:** 2015-06-15

**Authors:** Hanane Latrech, Imane Skikar, Mohammed El Hassan Gharbi, Abdelmjid Chraïbi, Ahmed Gaouzi

**Affiliations:** ^1^Department of Endocrinology, Mohammed VI Hospital, Medical School, Mohammed the First University, 60 000 Oujda, Morocco; ^2^Department of Radiology, Mohammed VI Hospital, Medical School, Mohammed the First University, 60 000 Oujda, Morocco; ^3^Department of Endocrinology, Ibn Sina Hospital, Medical School, Mohammed V University, 10 000 Rabat, Morocco; ^4^Department of Endocrinology Pediatrics, Children Hospital, Medical School, Mohammed V University, 10 000 Rabat, Morocco

## Abstract

*Background*. 47XYY syndrome is a rare sex chromosome variation characterized by an additional Y chromosome. Most patients with 47XYY karyotype have normal phenotype. This disorder seems associated with a higher risk of developing behavioral and cognitive problems, tall stature, and infertility in adulthood. Sexual development disorder is a rare finding. We report a first case with an abnormal left coronary artery originating from the pulmonary artery in a 47XYY patient. *Case*. A one-month-old child was referred for ectopic testis and micropenis. Physical examination revealed facial dysmorphia, micropenis, and curvature of the penis with nonpalpable testis. Laboratory tests showed decreased total testosterone and anti-Mullerian hormone (AMH) levels. Blood karyotyping revealed a 47XYY chromosomal formula. At the age of 3 months, the patient developed dyspnea and tachycardia. Echocardiography revealed an anomalous left coronary artery from pulmonary artery with left ventricular dysfunction requiring surgical revascularization by direct reimplantation of the left coronary artery system. Our second case was a 3-year-old child referred for hypospadias with nonpalpable left testicle. Physical examination showed hypertelorism. Blood karyotyping revealed a 47XYY chromosomal formula. *Conclusion*. To our knowledge, this is the first case of 47XYY syndrome associated with this congenital heart malformation and a sexual development disorder.

## 1. Introduction

47XYY variation is not an uncommon sex chromosome anomaly estimated in 1/1000 male births [1–3]. It is characterized by an additional Y chromosome secondary to a paternal nondisjunction at meiosis II. In some cases, the failure occurs in the cell division of the postzygotic mitosis in early embryonic development and produces a mosaic 46XY/47XYY karyotype [4–6]. Most patients with 47XYY karyotype have normal phenotype and consequently will have a delayed diagnosis. The child with this abnormality may develop behavioral problems, mild learning disability, delayed speech and language development, and tall stature [3]. We report two cases with 47XYY karyotype associated with micropenis, ectopic testis, and hypospadias rarely described in the literature, one of whom presents congenital heart defect.

## 2. Case 1

**Figure 1:**
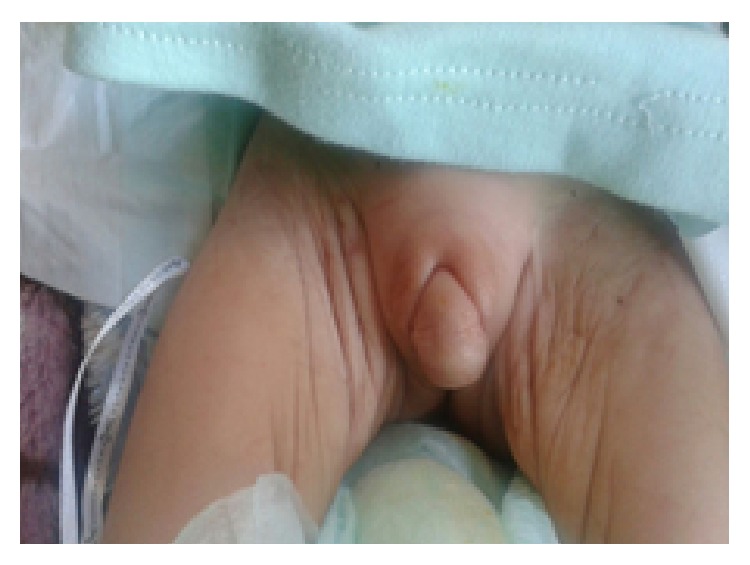
Case 1: testis ectopia, micropenis.

**Figure 2:**
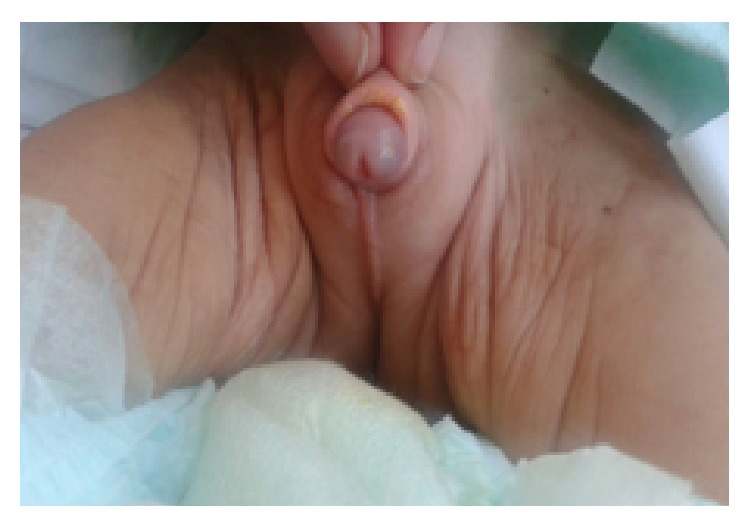
Case 1: curvature of the penis.

A one-month-old child was referred for ectopic testis and micropenis. In the family history, we found a history of sterility in the father aged 34 years linked to an oligoasthenospermia. There was no family history of consanguinity or similar cases. The patient was born at term with a normal birth weight and height. Physical examination revealed facial dysmorphia including hypertelorism, flat midface, and very marked philtrum. External genitalia examination showed a micropenis (1.5 cm) and curvature of the penis with nonpalpable testis (Figures 1 and 2). The rest of the somatic examination was normal. Laboratory tests showed an LH level at 3.9 UI/L (normal values: 0.55–5.5 mUI/mL), FSH at 7 UI/L (normal values: 1.3–7 mUI/mL), total testosterone level at 1.65 ng/mL (normal values: 1.8–4 ng/mL) and anti-Mullerian hormone (AMH) level at 13.20 ng/mL (normal values: 24–124 ng/mL (EIA Immunotech)). Pelvic ultrasonography showed the testis in inguinal position. Blood karyotyping revealed a 47XYY chromosomal formula. At the age of 3 months, hormonal evaluation showed a total testosterone level at 1.86 ng/mL. Also, the patient developed dyspnea and tachycardia with the presence of cardiomegaly apparent in a chest X-ray. Electrocardiography (ECG) detected q waves in leads DI and aVL with repolarization abnormalities in the left leads. Echocardiography revealed an anomalous left coronary artery originating from the pulmonary artery with left ventricular dysfunction requiring surgical revascularization by direct reimplantation of the left coronary artery system.

## 3. Case 2

**Figure 3:**
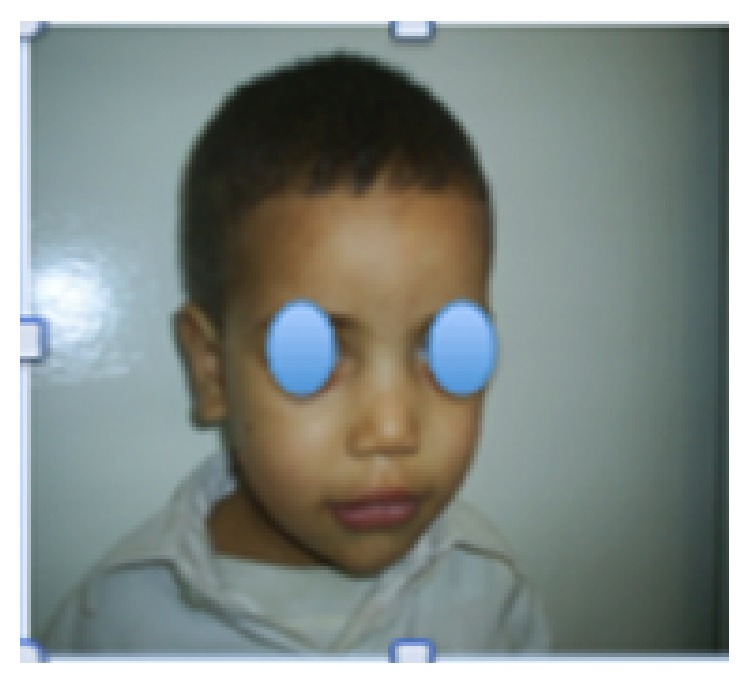
Case 2: hypertelorism.

**Figure 4:**
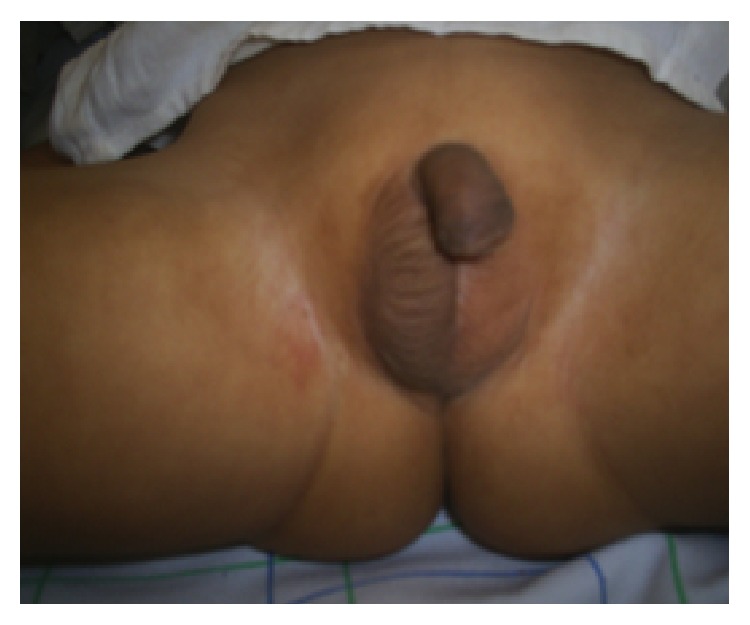
Case 2: nonpalpable left testicle and prepubertal right testicle in scrotal position.

**Figure 5:**
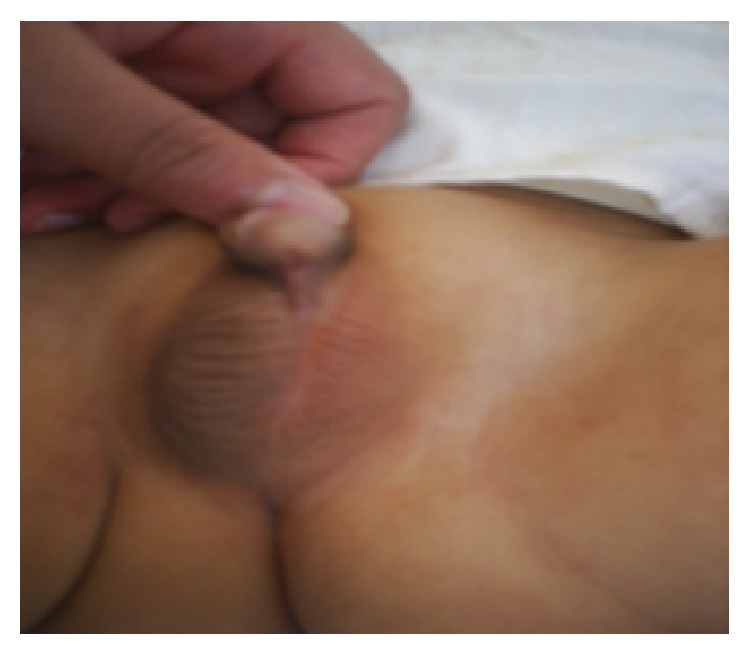
Case 2: hypospadias.

**Figure 6:**
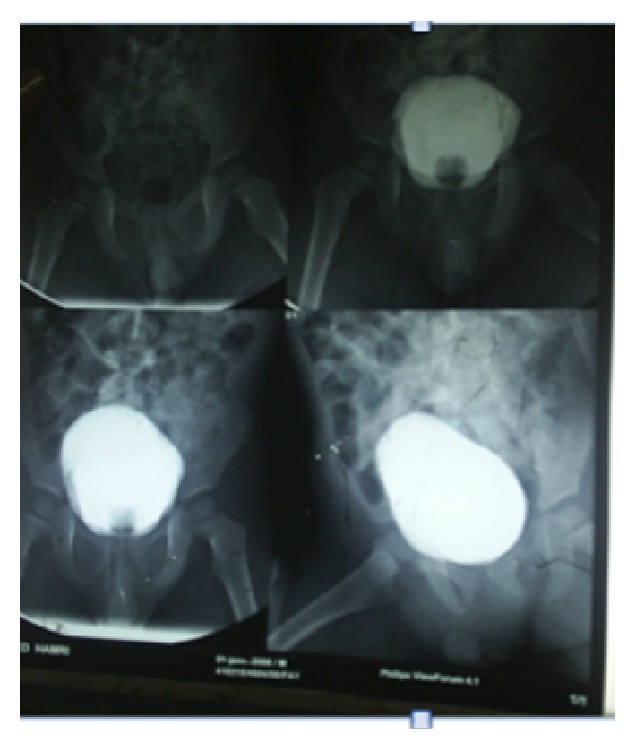
Case 2: male urethra.

A 3-year-old child was referred to our institution for undescended testes and hypospadias. There was no family history of consanguinity or similar cases. The patient was born at 41 weeks of gestational age with normal birth weight and height. Physical examination revealed normal stature and weight growth for the age, hypertelorism without other facial dysmorphia (Figure 3), and normal psychomotor development. There were no signs of tumor and no other signs of endocrinopathy. Neurological and systemic examinations were unremarkable. External genitalia examination showed anterior hypospadias with nonpalpable left testicle. The right testicle was prepubertal (<3 mL) in scrotal position (Figures 4 and 5). Serum FSH, LH, and testosterone results were age-normal. Both assay of AMH and hCG testing were prescribed. Pelvic ultrasonography showed absence of visible left testis. Genitography revealed a male urethra without Mullerian ducts (Figure 6).

The karyotype revealed chromosomal formula 47XYY. Surgical treatment for hypospadias and ectopic testis was proposed with medical monitoring of psychomotor and stature development and puberty.

## 4. Discussion

47XYY variation is the most common sex chromosome anomaly after Klinefelter syndrome (47XXY). The first case with this chromosomal disorder was described in 1961 by Sandberg et al. [7]. This anomaly was the last of the sex chromosome aneuploidies to be discovered. The separation of chromosomes during metaphase II of meiosis called nondisjunction may reveal germ cells with an extra copy of chromosome Y. If one of these atypical sperm cells is involved in fertilization, the child will have an extra Y chromosome in all cells. In some cases, the error occurs in the cell division of the postzygotic mitosis in early embryonic development. This can produce a mosaic 46, XY/47, XYY [3–6]. In 1970, some studies demonstrated that a higher number of persons with 47XYY were in penal and psychiatric institutions [8, 9]. Later, these studies were recognized as having significant methodological flaws [8]. It is currently reported that the patient with 47XYY karyotype has cognitive behavioral deficits including delayed language and motor development, impulsivity, poor attention span, and impairment in social interaction with increased risk of autism spectrum disorders [3, 8, 10–15]. In our second case, we did not find these anomalies at this age (3 years). A psychomotor evaluation was proposed with regular monitoring. The study of neuroanatomical variation in 47XYY has shown increased brain matter volumes, a finding putatively related to the increased frequency of autism spectrum disorders. In addition, frontotemporal grey and white matter reductions in XYY syndrome provide a likely neuroanatomical correlate for observed language impairments [10, 16, 17]. It has been reported as an association with 47XYY and migrating partial seizures in infancy, a very rarely reported epilepsy syndrome with unknown etiology. The mechanism of this association is not explained [18]. Most patients with this genetic disorder have no phenotypic abnormalities. Some authors reported tall stature beginning before puberty and increased head circumference [3, 8, 10–12, 19]. In this paper, we report this syndrome recognized at an earlier stage with presence of hypertelorism reported in 59% of cases with 47XYY and disorders of sexual development including hypospadias, micropenis, and ectopic testis. This association has been rarely reported [20–26] (Table 1) and the pathophysiology of the association is not clearly explained. The mechanism by which the excess of genes due to an additional Y chromosome can affect sexual development remains to be fully elucidated. Thus, the external genitalia examination in all patients with 47XYY karyotype may help the clinician to determine whether these disorders of sexual development comprise an incidental finding or are part of the spectrum of XYY syndrome which remains undetected. Some authors have investigated gonadic function in 47XYY children and adolescents and found normal or delayed puberty [26, 27]. Macroorchidism seems to be common with normal testosterone levels [26]. But there have also been reports of a normal or total absence of the testis [20, 28] and increased testosterone levels in institutionalized men with XYY karyotype, with normal [26, 29] or aggressive behavior [27]. In XYY patients with a disorder of sexual development, Rivera et al. reported age-normal basal plasma testosterone levels with an adequate response of the left testicular Leydig cells after hCG stimulation [20]. Our first case revealed a low peak of testosterone secretion (1.86 ng/mL) for a 3-month-old infant. As markers of testicular exocrine functions, inhibin B and anti-Mullerian hormone (AMH) levels were reported as normal in most of the cases. We reported low AMH levels in our first patient. A decrease in the levels of inhibin B has been described in some patients sometimes associated with a high FSH [26, 30]. This may explain some cases of infertility in adults with 47XYY. Indeed, the association with infertility problems was reported with scrotal findings ranging from normal to atrophic testicles, oligospermia, and varying endocrine profiles [3, 8, 10, 31]. It is currently known that some cases with infertility are associated with Yq microdeletions with a significant diagnostic and prognostic value. Therefore, some authors have suggested that, in XYY patients, the excess of copies of genes in the long arm of the Y chromosome could explain this infertility [26, 32]. The offspring of some patients with 47XYY have been studied, yielding reports of abnormal children with gonadal dysgenesis and Down's syndrome [33]. In the child with the extra chromosome, 21 came from the mother. Thus, the necessity of amniocentesis remains to be discussed. In Klinefelter syndrome, the most common genetic disorder characterized by an additional X chromosome, we find tall stature with impairments in language and motor ability similar to those observed in persons with 47XYY karyotype. In addition, Klinefelter syndrome manifests with hypogonadism symptoms including cryptorchidism, small testicles, gynecomastia, impuberism or incomplete pubertal development, sterility in adulthood, and shorter life spans compared with persons with normal karyotype. This shorter lifespan may be due to an increased risk of cancer, pulmonary, neurologic, and unspecified diseases. It has been noted that, for patients with 47XYY, there is significant delay of diagnosis, reduced life expectancy, and an increased overall and cause-specific mortality [3, 34, 35]. The spectra of clinical manifestations of these two different genetic entities vary and seem to produce partially overlapping phenotypes. Thus, multicenter studies are required to further investigate this unusual feature with more evaluation of gonadal function and long-term follow-up in individuals with 47XYY karyotype. In addition, our first patient had a congenital heart defect consisting of an anomalous left coronary artery originating from the pulmonary artery with left ventricular dysfunction requiring surgical reimplantation of the left coronary artery system. It is reported that up to 33% of congenital heart defects are associated with fetal aneuploidy and the majority of fetuses with congenital heart defect and aneuploidy also have extracardiac anomalies [36].

To our knowledge, this is the first case of 47XYY syndrome associated with a disorder of sexual development along with this congenital heart defect. This raises the questions, not only of the mechanism of this association but also of whether this defect is an incidental finding or a new part of the spectrum of XYY syndrome.

## 5. Conclusion

47XYY syndrome is the most common aneuploidies after Klinefelter syndrome. To date, men with 47XYY have been found to manifest with a characteristic physical phenotype including tall stature and cognitive behavioral deficits with increased risk of autism spectrum disorders and infertility in adulthood. We report a case with this sex chromosome variation associated with disorder of sexual development, an uncommon clinical phenomenon, and with congenital heart malformation. The mechanism of this association remains to be fully elucidated and it remains unclear exactly how an additional Y chromosome could affect testicular and cardiac development in 47XYY syndrome. Thus, multicenter studies are required to carry out more evaluation of gonadal and cardiac function and long-term follow-up in individuals with 47XYY karyotype.

## Figures and Tables

**Table 1:** Disorder of sexual development in a patient with 47XYY.

	*n*	Age	DSD
Rivera et al. 1979 [20]	1	5 10/12	Micropenis, hypospadias Right testis regression

Terada et al. 1984 [21]	1	29	Right undescended testis

Okamoto et al. 1988 [22]	2		Hypospadias, cryptorchism

Diego Nunez et al. 1992 [23]	2		Cryptorchism, puberty delay

Suzuki et al. 1999 [24]	1	11 months	Bilateral cryptorchism

Monastirli et al. 2005 [25]	1	72	Cryptorchism, gynecomastia

Bardsley et al. 2013 [26]	1/90 2/90 5/90		Hypospadias Cryptorchism Inguinal hernia

Our cases 2014	2	3 1 month	Hypospadias, ectopic testis Micropenis, ectopic testis (with congenital heart defect)
